# Recent advances in the chromatin-based mechanism of *FLOWERING LOCUS C* repression through autonomous pathway genes

**DOI:** 10.3389/fpls.2022.964931

**Published:** 2022-08-12

**Authors:** Jinseul Kyung, Myeongjune Jeon, Ilha Lee

**Affiliations:** ^1^School of Biological Sciences, Seoul National University, Seoul, South Korea; ^2^Research Center for Plant Plasticity, Seoul National University, Seoul, South Korea

**Keywords:** flowering, autonomous pathway, *FLOWERING LOCUS C*, histone modification, RNA polymerase II pausing, chromatin architecture, non-coding RNA

## Abstract

Proper timing of flowering, a phase transition from vegetative to reproductive development, is crucial for plant fitness. The floral repressor *FLOWERING LOCUS C* (*FLC*) is the major determinant of flowering in *Arabidopsis thaliana*. In rapid-cycling *A. thaliana* accessions, which bloom rapidly, *FLC* is constitutively repressed by autonomous pathway (AP) genes, regardless of photoperiod. Diverse AP genes have been identified over the past two decades, and most of them repress *FLC* through histone modifications. However, the detailed mechanism underlying such modifications remains unclear. Several recent studies have revealed novel mechanisms to control *FLC* repression in concert with histone modifications. This review summarizes the latest advances in understanding the novel mechanisms by which AP proteins regulate *FLC* repression, including changes in chromatin architecture, RNA polymerase pausing, and liquid–liquid phase separation- and ncRNA-mediated gene silencing. Furthermore, we discuss how each mechanism is coupled with histone modifications in *FLC* chromatin.

## Introduction

Proper timing of flowering, a phase transition from vegetative to reproductive development, is crucial for plant survival. Consequently, plants have evolved and developed various mechanisms to control flowering time in response to variable environments. Many plants in temperate regions have adopted winter-annual flowering traits that require prolonged cold winter temperatures for flowering in spring when the environment is favorable (Chouard, 1960; Bernier et al., 1993). However, some plants complete their life cycle rapidly, either in spring or fall (Weinig and Schmitt, 2004). For example, *Arabidopsis thaliana* accessions are classified into winter-annual and rapid-cycling types based on the requirement of long-term winter cold for rapid flowering (Michaels and Amasino, 2000). The underlying genetic difference in flowering traits between the two types is the presence or absence of the *FLOWERING LOCUS C* (*FLC*) and *FRIGIDA* (*FRI*) genes (Gazzani et al., 2003). *FLC,* which encodes a MADS-box transcription factor, acts as a potent floral repressor which inhibits the transcription of floral promoters, including *FT* (encoding florigen) and *SUPPRESSOR OF OVEREXPRESSION OF CONSTANS 1* (Lee et al., 2000; Michaels et al., 2005; Helliwell et al., 2006). FRI, a coiled-coil protein which forms part of a super protein complex, acts as a transcriptional activator of *FLC* (Michaels and Amasino, 1999; Choi et al., 2011; Li et al., 2018).

In rapid cyclers, the autonomous floral-promotion pathway (AP) induces early flowering by repressing *FLC* expression (Koornneef et al., 1998; Levy and Dean, 1998). Since *LUMINIDEPENDENS* (*LD*) was first isolated (Rédei, 1962; Lee et al., 1994), several genes, including *FCA*, *FLD*, *FLK*, *FPA*, *FVE*, and *FY*, have been cloned as AP genes (Macknight et al., 1997; Koornneef et al., 1998; Schomburg et al., 2001; He et al., 2003; Quesada et al., 2003; Simpson et al., 2003; Lim et al., 2004; Mockler et al., 2004). For the past two decades, researchers have investigated the biochemical functions of AP proteins. Reports suggest that a subset of AP proteins catalyze the epigenetic changes in *FLC* chromatin. Specifically, FVE and FLD constitute histone deacetylation or demethylation complexes, whereby the *FLC* chromatin turns into a repressive state (Liu et al., 2007; Yu et al., 2016). Additionally, several RNA-binding family proteins, such as FPA, FCA, and FY, indirectly repress *FLC* by mediating the 3′-end processing of *FLC* antisense transcript (Simpson et al., 2003; Liu et al., 2007; Hornyik et al., 2010; Liu et al., 2010). However, the function of AP proteins has yet to be completely understood.

Emerging evidence suggests that multiple layers of transcriptional processes determine transcript level (Gressel et al., 2019). In addition to well-known processes (e.g., enhancer- and histone modification-mediated gene regulation), regulatory mechanisms, such as RNA polymerase II (Pol II) pause–release control during transcriptional elongation and alternative polyadenylation during transcriptional termination, are critical gene regulatory processes (Tian and Manley, 2017; Chen et al., 2018). Notably, recent studies have consistently revealed that AP in floral promotion is also involved in such mechanisms. This review summarizes the latest findings on the molecular mechanisms of AP, including the control of chromatin architecture, Pol II pausing, and phase separation with ncRNA-mediated gene silencing.

## Architecture of *FLC* chromatin and AP

The 3D property of chromatin plays a vital role in transcriptional regulation (Dileep and Tsai, 2021; Deng et al., 2022). Although histone modifications such as methylation and acetylation have been the main focus of the studies for transcriptional regulation over the past two decades, studies on how chromatin architecture, such as chromatin loops, R-loops, and DNA topology, controls gene expression have been actively conducted in recent years (Kadauke and Blobel, 2009; Kouzine et al., 2014; Al-Hadid and Yang, 2016). Accordingly, the AP-mediated repression of *FLC* has been re-examined based on its chromatin architecture.

**Figure 1:**
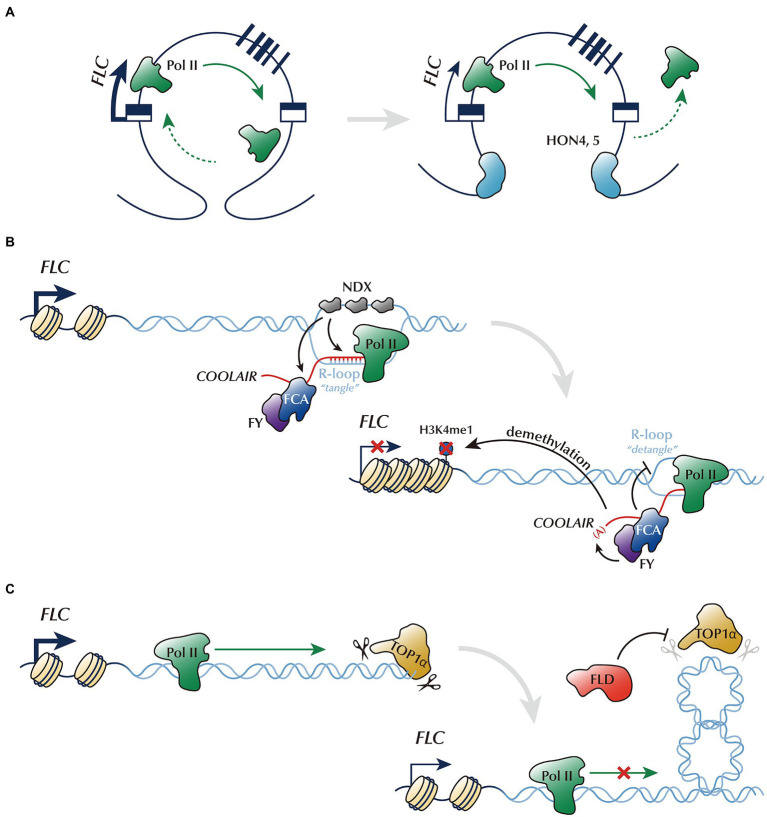
Control of *FLC* chromatin architecture by the autonomous pathway. **(A)** Chromatin loop linking the 5′- and 3′-ends of *FLC* will likely intensify the *FLC* transcription by promoting Pol II recycling. GH1-HMGA family proteins, HON4 and HON5, disrupt this chromatin looping, and thus reduce *FLC* expression. **(B)** NDX stabilizes the R-loop at the *FLC* 3′-end, where the antisense ncRNA, *COOLAIR*, is transcribed (tangled). This process probably enhances the binding of FCA/FY onto *COOLAIR* and inhibits Pol II progression. FCA/FY, in turn, represses *FLC* expression by promoting the proximal polyadenylation of *COOLAIR* and resolving the R-loop (detangled). **(C)** Antagonized function of TOP1α and FLD controlling DNA topology. TOP1α enhances *FLC* transcription potentially by reducing the torsional stress generated by DNA supercoiling. FLD partially counteracts TOP1α activity.

Chromatin loops, defined as the intergenic or intragenic bending of chromatin, are observed genome-wide in *Arabidopsis* (Grob et al., 2013; Feng et al., 2014). The 5′-end region of the *FLC* locus is connected to either the first intron or 3′-end region to form chromatin loops (Crevillén et al., 2013; Kim and Sung, 2017; Li et al., 2018). Importantly, the loop linking the 5′- and 3′-ends of *FLC* may contribute to *FLC* activation, possibly through enhancing Pol II recycling (Crevillén et al., 2013; Li et al., 2018). A recent study has identified novel AP members, the GH1-HMGA family proteins, which are involved in regulating this loop (Zhao B. et al., 2021). GH1-HMGA family proteins, also known as HIGH MOBILITY GROUP A4, 5 (HON4, 5), are homologs of human HMGA proteins which bend or unwind local chromatin structure (Ozturk et al., 2014). Similar to other AP mutant lines, the *honq* (*gh1-hmga quadruple*) mutant line exhibits increased *FLC* expression and delayed flowering (Zhao B. et al., 2021). Given that the *FLC* gene loop in the *honq* mutant line is increased, it has been suggested that the disruption of gene looping by GH1-HMGA family proteins may repress *FLC* expression by altering chromatin structures required for effective transcription (Figure 1A; Zhao B. et al., 2021). However, the causal relationship between the chromatin looping and the repression of *FLC* by GH1-HMGA family proteins should be validated in the future study. In contrast to the GH1-HMGA family proteins, the histone variant H3.3 appears to stabilize *FLC* looping by binding at both ends of *FLC* gene (Zhao F. et al., 2021). Importantly, *h3.3* knock-down mutants (*h3.3kd*) consistently show reduced *FLC* looping and decreased *FLC* level (Zhao F. et al., 2021). Therefore, it is likely that the opposite effects of GH1-HMGA family proteins and H3.3 for the *FLC* looping may be associated with their antagonistic function on *FLC* expression. BAF60, a component of the *Arabidopsis* SWI/SNF (SWITCH/SUCROSE NON-FERMNETABLE)-type ATP-dependent chromatin remodeling complex, also participates in *FLC* repression by affecting *FLC* gene looping (Jégu et al., 2014). It has been shown that the RNA interference lines of *BAF60* (*BAF60 RNAi*) display an increased number of *FLC* gene loops and upregulated expression of *FLC,* thereby producing the late-flowering phenotype in long days. This finding suggests that *BAF60* plays a negative role in loop formation. Histone modifications including H3K27me3, H3K9Ac, and H2A.Z replacement, are also altered in the *BAF60 RNAi* lines; thus, the effect of BAF60 on *FLC* gene looping may be mediated through histone modifications. One caveat is that *BAF60* is not a typical AP gene because the *BAF60 RNAi* lines do not show delayed flowering in short days. The increased *FLC* level caused by *BAF60 RNAi* is probably masked by the additional targets of *BAF60*. Therefore, *BAF60* may also be an *FLC* repressor which acts on gene looping. The functional interdependency between GH1-HMGA family proteins and BAF60 needs further analysis.

R-loops are another type of chromatin architecture which are composed of a DNA:RNA hybrid and an associated non-template single-stranded DNA (Al-Hadid and Yang, 2016). R-loops play important roles in gene expression, genome stability, and epigenomic signatures (Gao et al., 2021). *FLC* chromatin has an R-loop around its 3′-end, where the antisense transcript *COOLAIR* is transcribed (Sun et al., 2013; Baxter et al., 2021; Xu et al., 2021b). NODULIN HOMEOBOX (NDX) is a potential AP member that reportedly stabilizes this R-loop by binding onto the non-template ssDNA region (Sun et al., 2013). The increased *COOLAIR* level and the reduction of FCA-*COOLAIR* interaction in the *ndx* mutant suggest that R-loop stabilizing processes likely inhibit further transcription of *COOLAIR* and enhance binding of FCA onto *COOLAIR* (Xu et al., 2021b). The *fca* and *fy* mutants show an increased level of R-loops, suggesting that FCA and its binding partner, FY, act to resolve the R-loops (Figure 1B). Thus, R-loop dynamics, involving the stabilization by NDX and resolution by FCA and FY, result in *FLC* repression. However, the detailed mechanism by which R-loops participate in *FLC* transcription warrants further investigation. Furthermore, the loss of m^6^A methyltransferase (*mRNA ADENOSINE METHYLASE, MTA*) increases the level of R-loops, indicating that the N^6^-methyladenosine (m^6^A) modification of RNA is involved in R-loop resolution (Xu et al., 2021b). MTA interacts with FCA and is a potential AP member, as evidenced by the increased *FLC* expression in the *mta* mutant line. Moreover, a follow-up study showed that the resolution of the R-loop by FCA or FY is required for the proper progression of DNA replication fork, suggesting an interplay between DNA replication and transcription (Baxter et al., 2021).

AP is also possibly involved in regulating DNA topology. During transcription, torsional stress generated by DNA supercoiling inhibits proper transcription (Liu and Wang, 1987). Thus, the proper release of supercoiling by topoisomerases is required for transcriptional activation (French et al., 2011). Consistently, DNA topoisomerase I, TOP1α, in *Arabidopsis*, which binds to *FLC* chromatin, promotes *FLC* expression (Gong et al., 2017). Thus, the modulation of DNA topology by TOP1α promotes *FLC* transcription, possibly through Pol II accommodation. In contrast, the AP protein, FLD, counteracts TOP1α (Inagaki et al., 2021). FLD acts antagonistically to TOP1α for *FLC* transcription, as evidenced by the partial suppression of the late-flowering phenotype of *fld* in the *top1α fld* double mutant line (Gong et al., 2017). In addition, enhanced Pol II enrichment on the *FLD*-target genes in the *fld* mutant line is suppressed by *top1α* (Inagaki et al., 2021). This result suggests that FLD antagonizes the function of TOP1α and FLD is involved in the control of torsional stress on *FLC* chromatin (Figure 1C). However, the detailed function of FLD needs further elucidation.

## *FLC* repression by 3′-pausing of Pol II

During transcription in *Drosophila melanogaster*, or in mammalian cells, Pol II is transiently paused before it enters the elongation phase (Adelman and Lis, 2012; Chen et al., 2018). Controlling Pol II pause–release is possibly a core determinant of gene expression, considering that successful Pol II release into the productive elongation phase is required for the complete transcription (Core and Adelman, 2019). In most animal genes, Pol II pauses after transcribing short stretches (approximately 30–50 nts) of RNA from the transcription start site (TSS). Several pause-inducing factors, including DRB sensitivity-inducing factor (DSIF) and negative elongation factor (NELF), are known to stabilize the paused Pol II (Wu et al., 2003; Wu et al., 2005).

In contrast to animals, plants were thought to have different types of Pol II pausing, because they lack NELF proteins (Hetzel et al., 2016). However, Pol II pausing at the 5′-end is also observed in plants, although Pol II is usually stalled near the transcription termination site (TTS) of plant genes according to the studies using Global Run-On sequencing (GRO-seq) and plant native elongating transcript sequencing (plaNET-seq) methods (Hetzel et al., 2016; Zhu et al., 2018; Kindgren et al., 2020). *FLC* appears to be one of the 3′-paused genes, as it shows the typical characteristics: it has a relatively long gene length, it expresses antisense RNAs, and it is relatively close to its neighbor gene with the same orientation (Yu et al., 2019; Inagaki et al., 2021).

**Figure 2:**
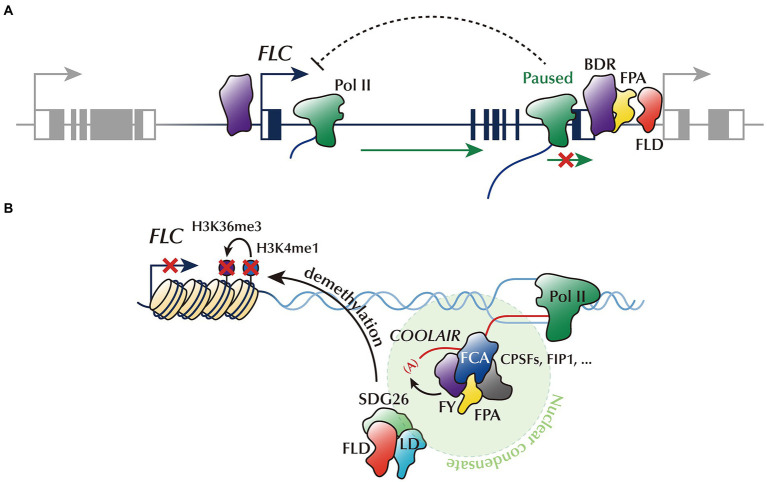
*FLC* repression by 3′-pausing, phase-separated AP proteins, and ncRNA. **(A)** AP proteins, BDR, FPA, and FLD, localizing at the gene borders, trigger 3’ Pol II pausing. Impediment of Pol II release into the elongation phase may reduce *FLC* transcript accumulation. **(B)** AP proteins, including FCA, FPA, FY, and RNA-processing factors, are condensed into phase-separated nuclear condensates to promote the proximal polyadenylation of *COOLAIR*. This phase-separated ncRNA-processing machinery transiently interacts with FLD/LD/SDG26, H3K4me1 demethylase complex, thereby removing the active histone marks (H3K4me1 and H3K36me3) from *FLC* chromatin.

Emerging evidence suggests that some AP genes act as pause-inducing factors which may govern *FLC* transcription (Figure 2A). For example, a recent study has identified novel AP members, called *BORDER* (*BDR*) family genes (Yu et al., 2021). Similar to other AP mutants, the *bdr123* triple mutant shows delayed flowering with elevated expression of *FLC*. In general, BDR proteins localize at the borders of genes close to their neighbor genes (Yu et al., 2019). They are likely to inhibit the progression of Pol II over the gene border, thereby preventing Pol II invasion into the promoters of downstream genes. However, the impediment of Pol II elongation by BDRs may result in decreased transcript accumulation. *FLC* may also be a target of such an inhibitory mechanism; however, further research is warranted for verification. Notably, the popular AP protein, FPA, is located at the borders of genes, especially at TTS (Yu et al., 2021). In addition, FPA physically interacts with BDR proteins and shares common targets. Therefore, it would be valuable to address whether FPA also promotes 3’ Pol II pausing in a similar manner to BDR, especially around the *FLC* locus. In addition, there is still uncertainty around whether 3’ Pol II pausing causes *FLC* gene silencing.

FLD, another major AP component, reportedly modulates Pol II pause–release (Inagaki et al., 2021). FLD is enriched at the TSS and TTS of genes rather than their gene body. Pol II is stalled around the TTS of FLD-targeted genes, and such 3’ Pol II pausing is conspicuously reduced in the *fld* mutant, indicating that FLD accelerates Pol II pausing. Since FLD occupies the 3′-end regions of *FLC* (Inagaki et al., 2021), *FLC* transcription is potentially repressed by FLD-promoted 3’ Pol II pausing. The physical interaction between FLD and LD proteins and similar transcriptome profiles between the *fld* and *ld* mutants suggest that FLD and LD cooperatively regulate the transcription (Fang et al., 2020; Inagaki et al., 2021). While the genome-wide function of FLD on 3’ Pol II pausing has been addressed (Inagaki et al., 2021), whether FLD also triggers 3’ Pol II pausing on the *FLC* locus is yet to be confirmed.

Accumulating evidence from studies using metazoans suggests an interplay between Pol II pausing and chromatin landscape. For instance, a rapid release of Pol II facilitates a broad distribution of active histone marks over the gene body, which is tightly linked with the high expression of the gene (Chen et al., 2015; Tettey et al., 2019). In contrast, Polycomb-group (PcG) proteins that catalyze the deposition of repressive histone marks preferentially target paused promoters (Enderle et al., 2011). Similarly, AP-mediated 3′-pausing at *FLC* may switch the *FLC* chromatin state inactive, thus suppressing *FLC* transcription. Consistent with this idea, 3’ Pol II pausing events triggered by the BDRs and FPA are correlated with the removal of H3K4me3 and the deposition of H3K27me3 (Yu et al., 2021). Furthermore, FLD and LD are likely to remove H3K4me1 from the gene bodies of their targets, suggesting that the AP proteins coordinate transcriptional events with chromatin silencing (Fang et al., 2020; Inagaki et al., 2021). Future research should explore the mechanism by which Pol II pause–release is linked to histone modifications for *FLC* suppression.

## Phase-separated AP proteins- and non-coding RNA-mediated gene silencing

Non-coding RNAs (ncRNAs) are RNAs that are not translated into proteins. They function in transcriptional or post-transcriptional gene regulation, structural organization of nuclear bodies, and genome integrity control (Ponting et al., 2009; Statello et al., 2021). The *FLC* locus also produces multiple long non-coding RNAs, such as *COOLAIR*, *COLDAIR*, and *COLDWRAP* (Swiezewski et al., 2009; Heo and Sung, 2011; Kim and Sung, 2017), all of which reportedly control dynamic alterations of chromatin state in the *FLC* locus after long-term cold exposure (Csorba et al., 2014; Kim and Sung, 2017; Kim et al., 2017; Zhao Y. et al., 2021). Among these RNAs, *COOLAIR*, an antisense transcript produced from the 3′-end of *FLC,* has been proposed to play a role in the epigenetic control of *FLC* with the help of AP proteins (Whittaker and Dean, 2017; Wu et al., 2020).

Multiple studies suggest that several AP genes, especially those encoding RNA-processing factors, control the 3′-end processing of *COOLAIR* (Hornyik et al., 2010; Liu et al., 2010; Marquardt et al., 2014; Wang et al., 2014). Some RNA-processing factors, such as a core spliceosome subunit [PRE-MRNA PROCESSING 8 (PRP8)] and a transcriptional elongation factor [CYCLIN-DEPENDENT KINASE C;2 (CDKC;2)], have been identified as AP members (Marquardt et al., 2014; Wang et al., 2014). The functions of *PRP8* and *CDKC;2* in *FLC* repression are dependent on *COOLAIR*; *prp8* or *cdkc;2* does not upregulate *FLC* expression any further if the *COOLAIR* promoter is replaced with *rbcs3B* terminator sequence [*FLC-TEX* in Marquardt et al. (2014) and Wang et al. (2014)]. Consistent with this, previous studies reported that PRP8 and CDKC;2 indirectly affect the expression of *FLC* by promoting the proximal polyadenylation of *COOLAIR* (Marquardt et al., 2014; Wang et al., 2014). The major AP genes, *FCA*, *FPA,* and *FY,* have also been proposed to control the processing of *COOLAIR.* FCA, FPA, and FY reportedly favor the usage of proximal poly(A) site in *COOLAIR* (Liu et al., 2010). Considering the epistatic interactions between *fca*, and *prp8* or *cdkc;2*, *FCA,* and *PRP8* or *CDKC;2* are thought to share the same genetic pathway to antagonize *FLC* expression (Marquardt et al., 2014; Wang et al., 2014).

Such *COOLAIR*-processing machinery is likely to be condensed into phase-separated nuclear bodies, and this process may be a mechanism behind *FLC* repression. FCA is clustered into nuclear condensates together with FPA, FY, and the subunits of polyadenylation machinery, including cleavage and polyadenylation factor 30 (CPSF30), CPSF100, and FH INTERACTING PROTEIN 1 (FIP1; Fang et al., 2019). FCA is required for the condensation of the polyadenylation machinery and directly associates with *COOLAIR* transcripts; thus, it likely concentrates the polyadenylation machinery near the *COOLAIR* to promote the usage of the proximal poly(A) site (Fang et al., 2019; Tian et al., 2019; Xu et al., 2021b). This condensation is enhanced by the prion-like domain (PrLD)-containing protein FLX-LIKE 2 (FLL2), RNA slicer ARGONAUTE 1 (AGO1), and m^6^A writer complex depositing m^6^A onto *COOLAIR* (Fang et al., 2019; Xu et al., 2021a,b).

The phase-separated *COOLAIR*-processing complex likely controls the *FLC* chromatin state through FLD. FLD assembles into a complex with LD and SET DOMAIN GROUP 26 (SDG26), which causes the removal of H3K4me1 deposited at *FLC* chromatin (Fang et al., 2020). This disables SDG8, which binds to H3K4me1 and facilitates the enrichment of H3K36me3, thereby suppressing *FLC* transcription (Fang et al., 2020). Recent results obtained using cross-linked nuclear immunoprecipitation and mass spectrometry (CLNIP-MS) suggest that a transient and dynamic interaction occurs between SDG26 and the components of the phase-separated poly(A) machinery, such as FCA, FPA, and FY (Figure 2B; Fang et al., 2019; Fang et al., 2020). In addition, AGO1, which is bound to *COOLAIR* at a proximal exon-intron junction region, also interacts with SDG26 (Xu et al., 2021a). Therefore, the *COOLAIR* 3′-processing event likely controls the *FLC* chromatin state through the physical interaction between components of the *COOLAIR* polyadenylation condensate and the FLD/LD/SDG26 protein complex. Moreover, this phase-separated polyadenylation complex, including FCA and FY, may resolve the *COOLAIR*-mediated R-loop at the 3′-end of *FLC* (Xu et al., 2021a,b), as mentioned earlier. Given that this R-loop is also closely connected to the histone modifications in other organisms (Chédin, 2016), this connection may be a missing link between co-transcriptional *COOLAIR* processing and *FLC* chromatin silencing. However, the causal relationship between *COOLAIR*-mediated R-loop processing and *FLC* chromatin silencing needs further verification (Xu et al., 2021b).

Recent studies have inferred that another clade of ncRNAs, small RNAs (sRNAs), could be associated with *FLC* repression. For example, AGO1 interacts with sRNA fragments that are complementary to *COOLAIR* (Xu et al., 2021a). Moreover, DICER-LIKE 1 (DCL1) and DCL3, required for sRNA production, are likely to suppress *FLC* independently of the FCA-mediated *FLC* silencing mechanism (Schmitz et al., 2007; Xu et al., 2021a). Therefore, a deeper understanding of the role of sRNAs in AP for flowering should be a focus in future research.

## Conclusion

This review summarizes the latest research progress in the autonomous pathway in *Arabidopsis*. Decades of studies have proposed unique mechanisms for *FLC* regulation, such as control of Pol II pause–release mechanism, modulation of chromatin architecture, and processing of ncRNA triggered by phase-separated machinery. The studies on such mechanisms are still in their infancy and heavily dependent on genome-wide transcriptome analyses. Thus, a large portion of the current models presented in this review has yet to be validated. Further verification of the proposed mechanisms through biochemical, genetic, and molecular work would be valuable to develop a better understanding of AP. In addition, this pathway has been closely linked with the epigenetic modification of *FLC* chromatin, particularly in relation to the changes in histone methylation patterns in the AP mutants. However, the detailed mechanism connecting the regulatory function of AP proteins described in this review and the epigenetic silencing of *FLC* remain largely unknown; thus, further studies are required.

The FRI complex strongly activates *FLC* expression even in the presence of AP proteins in winter-annual *Arabidopsis* (Johanson et al., 2000; Choi et al., 2011). This finding suggests that the *FLC* regulatory mechanisms of AP genes are counteracted by FRI complex. Therefore, there is a need for further studies elucidating the role of the FRI complex in the mechanisms of AP.

## Author contributions

JK and MJ drafted the manuscript. MJ prepared the figures. IL reviewed and edited the manuscript. All authors contributed to the article and approved the submitted version.

## Funding

This work was supported by the National Research Foundation of Korea (NRF) grant funded by the Korea Government (MSIT; No. NRF-2021R1A5A1032428).

## Conflict of interest

The authors declare that the research was conducted in the absence of any commercial or financial relationships that could be construed as a potential conflict of interest.

## Publisher’s note

All claims expressed in this article are solely those of the authors and do not necessarily represent those of their affiliated organizations, or those of the publisher, the editors and the reviewers. Any product that may be evaluated in this article, or claim that may be made by its manufacturer, is not guaranteed or endorsed by the publisher.
